# Jujuboside B suppresses angiogenesis and tumor growth via blocking VEGFR2 signaling pathway

**DOI:** 10.1016/j.heliyon.2023.e17072

**Published:** 2023-06-07

**Authors:** Pan Zhang, Xing Lai, Mao-Hua Zhu, Jiangpei Shi, Hong Pan, Yanhu Huang, Run-Jie Guo, Qin Lu, Chao Fang, Mei Zhao

**Affiliations:** a Department of Pharmacy, Shanghai University of Medicine & Health Sciences, Shanghai, 201318, China; b Hongqiao International Institute of Medicine, Tongren Hospital and State Key Laboratory of Systems Medicine for Cancer, Department of Pharmacology and Chemical Biology, Shanghai Jiao Tong University School of Medicine (SJTU-SM), Shanghai, 200025, China; c Key Laboratory of Basic Pharmacology of Ministry of Education & Joint International Research Laboratory of Ethnomedicine of Ministry of Education, Zunyi Medical University, Zunyi, 563003, China

**Keywords:** Jujuboside B, Triterpenoid saponin, Antiangiogenesis, Chick embryo chorioallantoic membrane, VEGFR2

## Abstract

Jujuboside B (JuB), one of the main active triterpenoid saponins from the traditional Chinese medicine *Ziziphus jujuba,* possesses a wide range of pharmacological activities. However, it is unknown whether JuB can inhibit tumor angiogenesis, a crucial step in solid tumor growth. In this study, we found that JuB significantly inhibited the proliferation, migration, and tube formation of human umbilical vein endothelial cells in a dose-dependent manner. JuB also suppressed angiogenesis in chick embryo chorioallantoic membranes and Matrigel plugs. Moreover, through angiogenesis inhibition, JuB delayed the growth of human HCT-15 colorectal cancer xenograft in mice. Western blot assay demonstrated that JuB inhibited the phosphorylation of VEGFR2 and its key downstream protein kinases, such as Akt, FAK, Src, and PLCγ1. In conclusion, the antiangiogenic potency and molecular mechanism of JuB are revealed for the first time, indicating that this triterpene saponin may be further explored as a potential drug candidate or lead compound for antiangiogenic cancer therapy.

## 1 Introduction

**Fig. 1:**
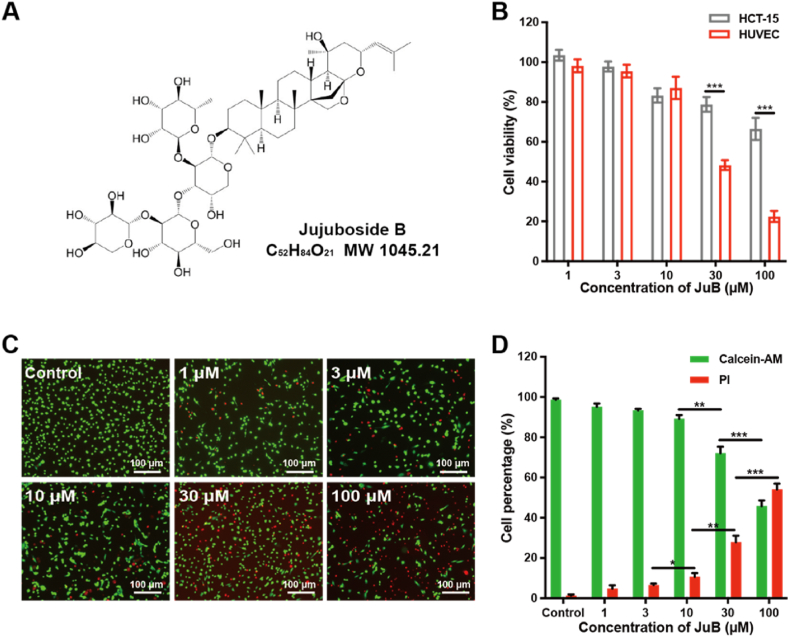
JuB suppressed the viability of HUVECs. (**A**) The chemical structure of JuB. (**B**) Effect of JuB on cell viability. (**C**) Representative photographs of JuB-treated HUVECs stained with calcein-AM/PI (Green: live cells; Red: dead cells). (**D**) The percentages of live (calcein^+^) and dead (PI^+^) HUVECs after JuB treatments. All values were presented as mean ± s.d. n = 3, *p < 0.05, **p < 0.01, ***p < 0.001. (For interpretation of the references to color in this figure legend, the reader is referred to the Web version of this article.)

Jujuboside B (JuB, Fig. 1A), a triterpenoid saponin extracted from *Ziziphus jujuba*, possesses various pharmacological activities, such as anti-inflammatory, antiplatelet, antianxiety, and antitumor effects [[1], [2], [3]]. The antitumor properties of JuB are exerted through a variety of molecular pathways. Specifically, JuB induces apoptosis of tumor cells through FasL and caspase-8 activation [4], caspase-3 activation [1], or NOXA upregulation [5]. However, the effect of JuB on tumor angiogenesis, a crucial step in solid tumor growth, remains unclear.

Angiogenesis is a complicated and well-ordered process that requires several signaling pathways to cooperate [6]. Among them, the vascular endothelial growth factor (VEGF) signaling pathway plays a vital function [7,8]. Inhibition of the VEGF signaling pathway has been demonstrated to have clinical benefits in cancer therapy [9,10]. So far, FDA has approved several antiangiogenic inhibitors targeting VEGF pathway, of which bevacizumab is the first antiangiogenic drug for colorectal cancer treatment [11]. Although the approved antiangiogenic drugs targeting VEGF pathway can improve survival of some cancer patients, their limited efficacy and side effects restrict their wide application [[12], [13], [14]]. The development of new antiangiogenic drugs with low toxicity, low cost, and good efficacy has become an urgent need. Recent studies have shown that natural products, especially Chinese herbal medicines with a long history of clinical application, have shown potent antiangiogenic activity with an acceptable toxic profile [15].

In this work, the effect and molecular mechanism of JuB in regulating tumor angiogenesis were investigated. JuB suppressed the proliferation, migration, and tube formation in human umbilical vein endothelial cells (HUVECs). JuB also displayed the antiangiogenic ability in chick embryo chorioallantoic membrane (CAM) and Matrigel plug models. JuB inhibited angiogenesis and tumor growth in the HCT-15 human colorectal cancer xenograft model. Furthermore, Western blot assay revealed that the underlying antiangiogenic mechanism of JuB is the blockade of VEGFR2 signaling pathway.

## 2 Materials and methods

### 2.1 Materials, cells, and animals

JuB (C_52_H_84_O_21_, purity >99%) was purchased from Push Bio-Technology Company. Fetal bovine serum (FBS), Trypsin-EDTA Solution, RPMI 1640 medium, penicillin, and streptomycin were provided by Basal Media Technologies. The VascuLife VEGF Cell Culture Medium and recombinant human vascular endothelial growth factor (rhVEGF_165_) were obtained from Lifeline Cell Technology. Phosphatase inhibitor cocktail, Protease Inhibitor Cocktail, and RIPA Lysis Buffer were obtained from Sangon Biotech. *Anti*-VEGFR2 (#2479), *anti*-pVEGFR2 (#2478), *anti*-PLCγ1 (#2822), *anti*-pPLCγ1 (#8713), *anti*-FAK (#3285), *anti*-pFAK (#8556), *anti*-Src (#2108), *anti*-pSrc (#59548), *anti*-Akt (#4685) and *anti*-pAkt (#4060) were supplied by Cell Signaling Technology. HRP-conjugated goat anti-mouse IgG H&L (#A0216), HRP-conjugated goat anti-rabbit IgG H&L (#A0208), and prestained protein marker (#P0072) were obtained from Beyotime Biotechnology. The LIVE/DEAD cell viability/cytotoxicity kit (#40747ES76) and BCA Protein Assay Kit were obtained from Yeasen Biotechnology.

The HCT-15 human colorectal adenocarcinoma cell line was purchased from American Type Culture Collection and cultivated in RPMI 1640 medium supplemented with 10% FBS and 1% penicillin/streptomycin. Primary HUVECs were obtained from Lifeline Cell Technology and cultured in the VascuLife VEGF Cell Culture Medium supplemented with 2% FBS and rhVEGF LifeFactor (5 ng/mL). Both HUVECs and HCT-15 were cultured in a humidified condition with 5% CO_2_ at 37 °C.

Female BALB/c nude mice were obtained from the Shanghai Laboratory Animal Center (Chinese Academy of Sciences, Shanghai, China). The animal experiments of this study were approved by the Ethical Committee of Shanghai Jiao Tong University School of Medicine (approval NO.: IACUC-A2018026) on November 23, 2018.

### 2.2 Cell viability assay

The effect of JuB on cell viability was examined by Cell Counting Kit-8 (Dojindo Laboratories, Kumamoto, Japan). HUVECs (6 × 10^3^ cells/well) or HCT-15 cells (6 × 10^3^ cells/well) were seeded into 96-well plates (Corning) and incubated at 37 °C for 12 h. When the cell confluence reaches 70%, the cells were treated with various concentrations of JuB (1, 3, 10, 30, and 100 μM) for 48 h. Subsequently, 10 μL CCK-8 solution was added to each well and the cells were incubated at 37 °C for 2 h. All groups were set for three replicate wells. Finally, the absorbance at 450 nm was detected with a microplate reader. Cell viability (%) was calculated based on the control.

The LIVE/DEAD cell viability/cytotoxicity kit was also used to evaluate the HUVEC viability. Briefly, HUVECs (1 × 10^4^ cells/well) were seeded in 96-well plates and cultured for 12 h. Then the cells were incubated with various concentrations of JuB (1–100 μM). After 48 h, HUVECs were replaced with PBS containing 2 μM calcein-AM and 4.5 μM propidium iodide (PI) for 15 min to evaluate cell viability. The live cells were marked in green (calcein-AM; Ex 490 nm, Em 515 nm), while the dead ones were marked in red (PI; Ex 535 nm, Em 615 nm).

### 2.3 Cell cycle assay

HUVEC cells were inoculated in a 6-well culture dish. After incubation with JuB (0–100 μM) for 24 h, the cells were harvested and then immobilized in 70% ethanol at 4 °C for 12 h. The cells were incubated with PBS containing RNase (10 μg/mL) and propidium iodide (50 μg/mL) for 30 min, then detected using the flow cytometer (Cytomics FC500, Beckman Coulter).

### 2.4 Wound healing assay

HUVECs were seeded in a 96-well plate (three replicates per group) at a density of 1 × 10^4^ cells/well. Then, the Wound Maker (IncuCyte) scratched the HUVECs. The cells were then washed with PBS and cultured with various concentrations of JuB (1–100 μM) for 12 h. Then the cells were incubated in fresh medium till 48 h, and the cells were photographed using the IncuCyte Live-Cell Analysis System (Essen BioScience). The closure area of the wound was quantified using Image-Pro Plus 8.0 software (Media Cybernetics, Bethesda, MD).

### 2.5 Transwell migration assay

The cell migration assay was also performed in a 24-well Transwell Boyden chamber with a polycarbonate filter of a pore size of 8 μm and 6.5 mm diameter inserts (Corning Costar, MA). Firstly, 5 × 10^5^ cells/well were suspended in a serum-free medium with various concentrations of JuB (1–100 μM) and seeded into the upper chamber of transwell 24-well plates. Then, the lower chamber was added with a 600 μL completed medium (containing 20 ng/mL VEGF_165_). The cells were cultured in an incubator (37 °C with 5% CO_2_). After 10 h, the non-migrating cells on the upper surface of the membranes were gently removed. Then the migrated cells were fixed in 4% glutaraldehyde for 20 min and stained with 0.5% crystal violet overnight at room temperature. After washing the membrane with PBS, the cells were photographed using an EVOS inverted microscope (Life Technologies, Grand Island, NY). The migrated cells were quantified with Image-Pro Plus 8.0 software.

### 2.6 Tube formation assay

Briefly, thawed Matrigel was coated at 70 μL/well in a pre-chilled 96-well plate and incubated at 37 °C for 10 min. Then the cells were seeded on the Matrigel at a concentration of 2 × 10^4^ cells/mL and treated with various concentrations of JuB (1–100 μM). After 8 h, the tubule development was imaged with an EVOS microscope (Life Technologies, Grand Island, NY), and analyzed using Image-Pro Plus 8.0 software.

### 2.7 Chick embryo chorioallantoic membrane assay

CAM assay was used to evaluate the antiangiogenic activity of JuB. Fertilized chicken eggs were pre-incubated at 37 °C in 60% humidity. After 7 days of incubation, a 1 cm^2^ small window was cut on the broad side of the eggs and then removed the shell membrane. A sterilized 5-mm diameter Whatman filter sheet soaked with JuB (1–100 μM) was put on the CAM (n = 3 in each group). Vehicle (Saline) alone was included as the control. The window was then sealed with parafilm and the eggs were incubated at 37 °C in 60% humidity for 48 h. The pictures of CAM were photographed, and the neovascularization was quantified by Image-Pro Plus 8.0 software.

### 2.8 Matrigel plug assay

400 μL Matrigel containing different concentrations of JuB (0, 10, 30, and 100 μM) with heparin (30 U/mL) and recombinant human VEGF_165_ (50 ng/mL) was subcutaneously injected into the flanks of female BALB/c mice. The Matrigel plugs (n = 3 in each group) were collected after 12 days and performed for CD31 immunofluorescence staining. Images of microvessels were photographed using an EVOS microscope. The microvessel density was quantified with Image-Pro Plus 8.0 software. The hemoglobin content of the Matrigel plugs was determined using a hemoglobin assay kit (#RC21550-50T, AMEKO, Shanghai, China).

### 2.9 Anticancer therapy of JuB in subcutaneous HCT-15 tumor in mice

For the investigation of the in vivo antitumor effect of JuB, HCT-15 cells (2 × 10^5^ cells) were subcutaneously inoculated on the right flanks of female BALB/c nude mice. When the average tumor volume reached approximately 80 mm^3^, the mice were randomly divided into two groups (each group n = 6). The mice were intraperitoneally injected with JuB (20 mg/kg) once every two days for 7 times. The tumor volume and body weight were recorded, and tumor volumes were calculated by: volume = (length × width^2^)/2. Mice were sacrificed on day 15, and the tumors were resected and weighed. Then paraffin-embedded tissue samples were sectioned for pathological examination. Then the sections were stained with rat anti-mouse CD31 antibody (1: 200, BD Biosciences, Shanghai, China) and *anti*-Ki67 rabbit pAb (1:500, Servicebio, Wuhan, China). Imaging was performed using a photomicroscope (Leica DFC 320) or confocal laser scanning microscope (Leica TCS-SP8). The images were quantitated by Image-Pro Plus 8.0.

### 2.10 Western blot analysis

In brief, HUVECs were seeded into 6-well plates (2 × 10^5^ cells/well) and incubated with a VascuLife medium until cell confluence reached 80%. Subsequently, the cells were treated with various concentrations (0–100 μM) of JuB for 30 min and stimulated with VEGF_165_ (50 ng/mL) for 4 min. Then the cells were extracted by RIPA solution which was supplemented with PMSF and the phosphatase inhibitor cocktail. The BCA Protein Quantification Kit was used to determine the concentration of protein. An equal amount of protein (30 μg/well) was applied to SDS-PAGE and transferred to the PVDF membrane (Millipore, Bill-erica, MA). The membrane was blocked with 5% non-fat milk for 1 h at room temperature. Finally, the membrane was incubated with primary antibodies (1:1000) overnight at 4 °C and then followed by incubation with the HRP-conjugated secondary antibodies (1:5000) for 2 h at room temperature. After washing the membrane three times with TBST, immunoreactive bands were visualized using the Enhanced ECL Chemiluminescent Substrate Kit (Yeasen, Shanghai, China) according to the manufacturer's instructions.

### 2.11 Statistical analysis

All data were analyzed as mean ± s.d. of at least three independent experiments. Significance between experimental groups was determined by one-way ANOVA with the Bonferroni multiple comparison post-test or Student's *t*-test. The *p* value below 0.05 was considered significant.

## 3 Results

### 3.1 JuB suppressed the viability of HUVECs more effectively than HCT-15

To evaluate the ability of tumor angiogenesis in vitro, HUVEC is used as a normal cell line to mimic the tumor-associated endothelial cells [16]. JuB significantly inhibited the viability of HUVECs in a dose-dependent manner (Fig. 1B). Low concentrations of JuB (1–10 μM) exhibited almost no effect on HUVEC viability, but when the concentration of JuB exceeded 30 μM, cell viability was significantly suppressed. Similar results were observed in the calcein-AM and PI dual staining assays (Fig. 1C and D). To examine whether the inhibitory effect of JuB on HUVEC viability was cytostatic or cytotoxic, we investigated the effects of JuB on the cell cycle of HUVEC. The percentage of cells in G0/G1 and S phases changed dramatically with JuB in a dose-dependent manner, indicating that JuB may inhibit HUVEC viability by arresting the G0/G1 phase (Fig. S1). In contrast, more than 65% of viabilities of HCT-15 cells were maintained after treatment with 1–100 μM JuB. It indicated that HUVECs were more sensitive to JuB than HCT-15, which implies that it may inhibit tumor growth through a specific antiangiogenic effect.

### 3.2 JuB inhibited migration and tube formation of HUVECs

**Fig. 2:**
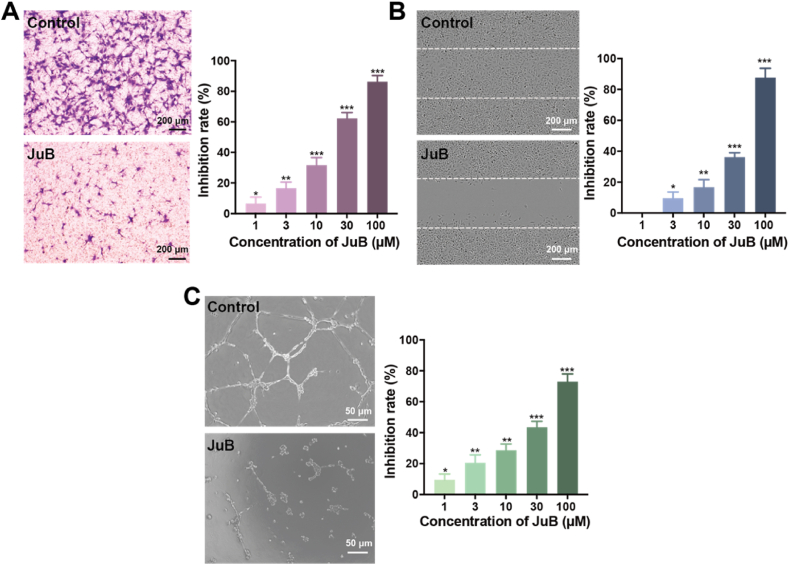
JuB significantly inhibited migration and tube formation of HUVECs. (**A**) HUVEC migration was suppressed by JuB in the Transwell assay. (**B**) JuB inhibited the horizontal migration of HUVECs in the wound healing assay. Imaging was performed with IncuCyte Live-Cell Analysis System. (**C**) JuB suppressed HUVEC tube formation. HUVEC tube formation was photographed and quantified using Image-Pro Plus 8.0 software. The photographs of the control and JuB groups (100 μM) were displayed in each panel. All values are presented as mean ± s.d. n = 3, *p < 0.05, **p < 0.01, ***p < 0.001.

Endothelial cell migration is an important procedure in angiogenesis and tumor growth [17]. We used the Transwell assay to investigate the effects of JuB on endothelial cell migration. As shown in Fig. 2A, after being treated with JuB for 10 h, the migrated HUVECs significantly decreased with increasing JuB concentration, indicating that JuB inhibited HUVEC migration in a dose-dependent manner. After 3 μΜ JuB treatment, ∼20% cell migration was inhibited. The inhibition rate of cell migration increased to approximately 90% when HUVECs were treated with 100 μΜ JuB. The wound healing assay was also used to evaluate the effects of JuB on HUVEC migration (Fig. 2B). After 48 h incubation, ∼20% horizontal migration inhibition was obtained at 10 μΜ JuB. More than 80% of cell migration was suppressed by 100 μM JuB. These findings demonstrated that JuB can effectively inhibit endothelial cell migration.

Endothelial cells can spontaneously form a tubular network structure on Matrigel, which can be used for in vitro angiogenesis assay. JuB dramatically inhibited HUVEC tube formation at concentrations from 1 to 100 μM (Fig. 2C, Fig. S2). Specifically, cells were individually dispersed on Matrigel with very few intercellular contacts after treatment with 100 μΜ JuB.

### 3.3 JuB inhibited angiogenesis in CAM

**Fig. 3:**
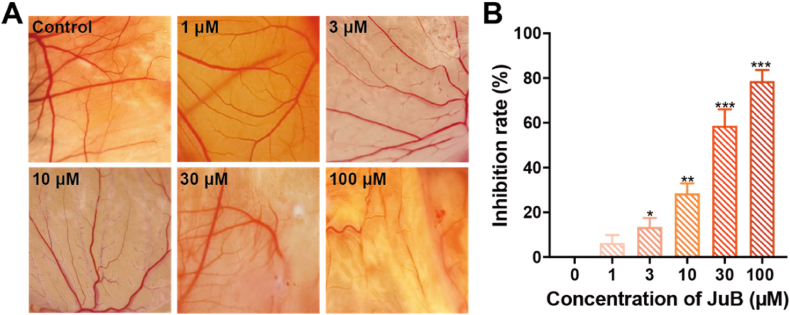
Antiangiogenic effect of JuB (1–100 μM) in the CAM. The representative images of new blood vessels were photographed (**A**) and quantified (**B**). The values are presented as mean ± s.d. n = 3, *p < 0.05, **p < 0.01, ***p < 0.001.

The CAM model was used to investigate the antiangiogenic effect of JuB in vivo [18]. JuB dose-dependently decreased the formation of new blood vessels in comparison to the control group (Fig. 3A and B). ∼80% blood vessels were suppressed at the highest test concentration (100 μM).

### 3.4 JuB inhibited vascularization in Matrigel plugs

**Fig. 4:**
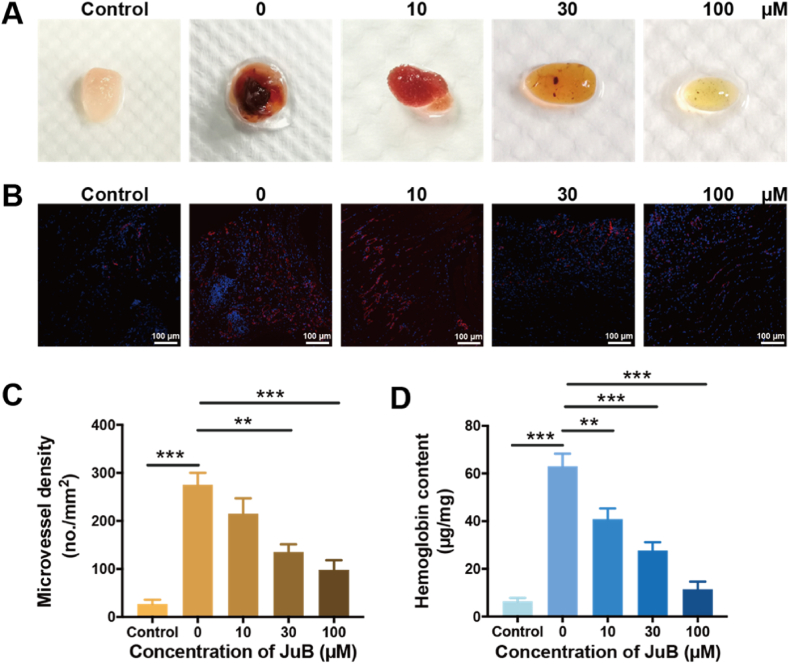
Effect of JuB on angiogenesis in the Matrigel plug. The Matrigel plugs were harvested 12 days post implantation. Matrigel without VEGF and JuB was set as control. (**A**) Representative photographs of the Matrigel plugs on day 12. (**B**) Immunofluorescence staining of CD31 in the Matrigel plugs. (**C**) Quantified microvessel density in panel B. (**D**) Hemoglobin content in Matrigel plugs. All values were shown as mean ± s.d. n = 3, *p < 0.05, **p < 0.01, ***p < 0.001.

The Matrigel plug was used to investigate the effect of JuB on angiogenesis in vivo. JuB dose-dependently suppressed the angiogenesis in Matrigel plugs, which can be confirmed from both the photographs and CD31-stained immunofluorescence micrographs (Fig. 4A, B, C). Hemoglobin contents in the Matrigel plug also reflected the decreased angiogenesis with the increase of JuB concentrations (Fig. 4D).

### 3.5 JuB suppressed angiogenesis and growth of HCT-15 tumors in mice

**Fig. 5:**
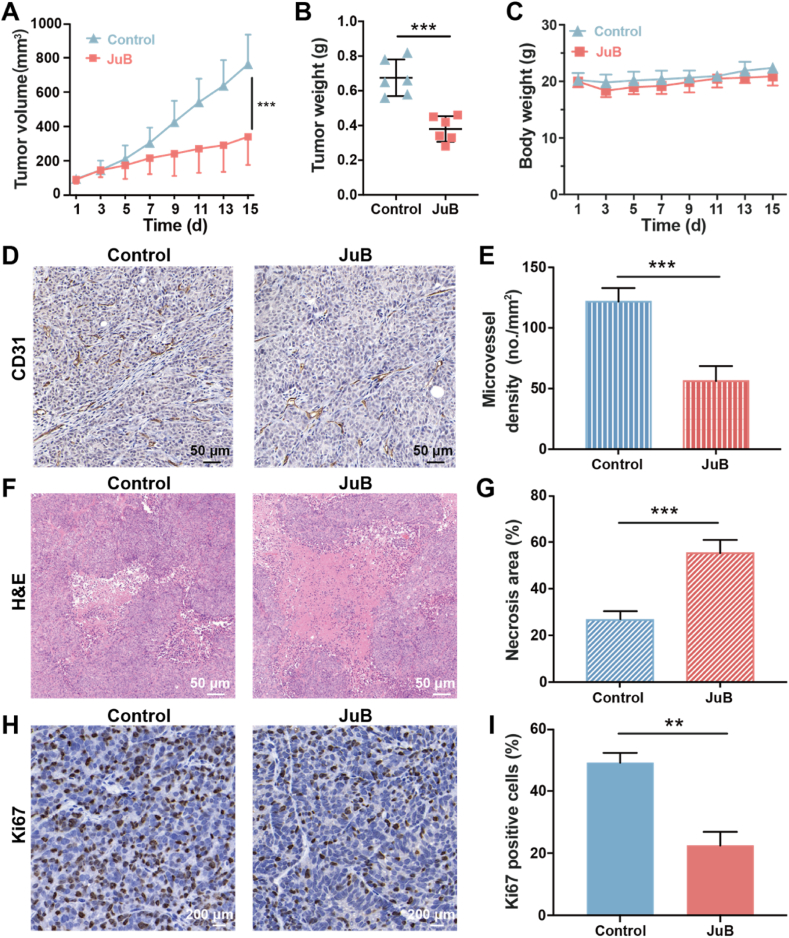
JuB inhibited tumor growth through antiangiogenic activity in a subcutaneous HCT-15 tumor model. (**A**) Tumor growth curve. (**B**) Tumor weight on day 15. (**C**) Body weight of mice. (**D**) Representative photographs of CD31-positive staining. (**E**) Quantified tumor microvessel density. (**F**) H&E staining sections of necrosis area. (**G**) Statistical analysis of necrosis area. (**H**) Proliferative tumor cells were stained by the Ki67 antibody. (**I**) Statistical analysis of Ki67-positive tumor cells. All values were shown as mean ± s.d. **p < 0.01, ***p < 0.001.

We hypothesized that JuB may inhibit tumor growth by suppressing angiogenesis, as it showed significant antiangiogenic properties in CAM and Matrigel plug models. The human HCT-15 colorectal cancer xenograft model was used to examine the hypothesis. JuB significantly inhibited the tumor growth compared to the control group, as illustrated in Fig. 5A. At the end of the test (day 15), the tumor volume with JuB treatment was 339.8 mm^3^, 55.5% less than in the control group (763.1 mm^3^). Meanwhile, tumor weights treated by JuB were 56.3% smaller than that of the control group (Fig. 5B). JuB treatment did not cause the loss of mouse body weight (Fig. 5C), indicating the good tolerance or low toxicity of the therapeutic regimen. To confirm whether the antitumor efficacy was related to the antiangiogenic activity, pathological and immunohistochemical assays of the HCT-15 tumor tissues were performed. JuB treatment resulted in pronounceably decreased microvessel density (Fig. 5D and E) and Ki67-positive cells (Fig. 5H and I), and significantly increased necrosis area (Fig. 5F and G) compared to the control group. These observations indicated that angiogenesis inhibition was involved in the antitumor effect of JuB in vivo, while the direct cytotoxicity of JuB to tumor cells (Fig. 1B) may also contribute to the delay of tumor growth.

### 3.6 JuB suppressed angiogenesis by blocking VEGFR2 signaling pathway and its downstream proteins

**Fig. 6:**
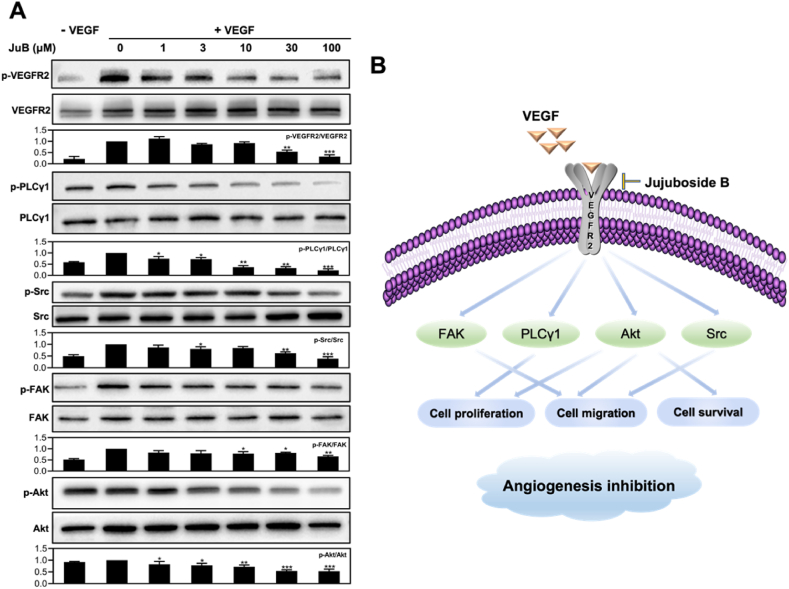
JuB inhibited angiogenesis via the VEGFR2 signaling pathways. (**A**) JuB suppressed the activation of VEGFR2 and its downstream signaling mediators (FAK, PLCγ1, Akt, and Src) in HUVECs. (**B**) Schematic diagram of JuB-mediated antiangiogenic signaling pathway. All values were shown as mean ± s.d. *p < 0.05, **p < 0.01, ***p < 0.001.

To reveal the underlying mechanism of the antiangiogenic effects of JuB in HUVECs, we evaluated the involvement of VEGFR2 and its downstream signaling pathway, which has an essential effect on angiogenesis by regulating endothelial cell function [19]. As shown in Fig. 6A and Fig. S3, Western blot assay showed that JuB inhibited the phosphorylation of VEGFR2 and its downstream signaling proteins, including FAK, PLCγ1, Akt, and Src, in a dose-dependent manner.

## 4 Discussion

Antiangiogenic therapy is a validated approach for clinic cancer treatment [20]. More than 10 drugs that target the VEGF/VEGFR axis have been approved by the FDA to treat a variety of tumors [21]. However, the clinical advantages are limited by treatment-related resistance, adverse effects, and high cost [13,21,22]. Thus, it will be beneficial to explore and develop novel antiangiogenic agents from natural products [23,24], which potentially have the advantages of low cost and toxicity.

JuB, a triterpenoid saponin derived from *Ziziphus jujuba*, exhibits antitumor effects that have been widely mentioned. However, it is unknown if JuB can inhibit tumor development via antiangiogenesis. In this study, we investigated the antiangiogenic activity of JuB. It was demonstrated that JuB had a dose-dependently inhibitory effect on angiogenesis-related activities of HUVECs, including proliferation, migration, and tube formation. In the in vivo models of CAM and Matrigel plugs, JuB also displayed excellent antiangiogenic effects. Notably, the significant antiangiogenic activity resulted in strong antitumor effects. JuB (20 mg/kg) resulted in over 50% tumor suppression in the subcutaneous HCT-15 colorectal cancer. Moreover, no significant weight loss was observed after JuB treatment. Biochemical parameters of hepatotoxicity and nephrotoxicity and differential assays for routine RBC (red blood cells) and WBC (white blood cells) were further used to evaluate treatment-related toxicity. No significant differences were observed in the JuB group compared to the control group, indicating a good tolerance of JuB treatment (Fig. S4).

Inhibiting the VEGFR-2 signaling pathway has been utilized as an effective therapeutic strategy in antiangiogenic therapy [25]. Western blot analysis revealed that VEGF-stimulated VEGFR2 phosphorylation and its downstream signaling mediators, including Src, FAK, Akt, and PLCγ1, were inhibited by JuB in a dose-dependent manner. FAK and Src regulate angiogenesis by modulating endothelial cell motility and proliferation [26]. Akt regulates the function of multiple downstream proteins that are involved in cell proliferation, migration, and survival [27]. PLCγ1 plays a vital role in VEGFR2-dependent MAPK signaling [28]. The antiangiogenic molecular mechanism of JuB is illustrated in Fig. 6B. Other triterpenoid natural products have also been found to possess antiangiogenic effects by blocking VEGFR2 signaling, such as Ailanthus excelsa chloroform extract-1 (AECHL-1) [29] and pristimerin [30]. The investigation of the structure-activity relationship will further help elucidate the mechanism of action of this natural compound.

In conclusion, our study demonstrated for the first time that JuB efficiently suppresses tumor angiogenesis by decreasing the activation of VEGFR2 and its downstream signaling pathways. These findings indicated that JuB can be further explored as a potential drug candidate or lead compound for antiangiogenic cancer therapy.

## Data availability statement

Data will be made available on request.

## Author contribution statement

Pan Zhang: Performed the experiments; Analyzed and interpreted the data; Wrote the paper. Xing Lai: Performed the experiments; Analyzed and interpreted the data; Wrote the paper. Maohua Zhu: Contributed reagents, materials, analysis tools or data. Jiangpei Shi: Performed the experiments; Contributed reagents, materials, analysis tools or data. Hong Pan: Contributed reagents, materials, analysis tools or data. Yanhu Huang: Contributed reagents, materials, analysis tools or data. Runjie Guo: Contributed reagents, materials, analysis tools or data. Qin Lu: Contributed reagents, materials, analysis tools or data. Chao Fang: Conceived and designed the experiments; Analyzed and interpreted the data; Wrote the paper. Mei Zhao: Conceived and designed the experiments; Wrote the paper.

## Declaration of competing interest

The authors declare that they have no conflict of interest.
