# Supplementary material for: Jujuboside B suppresses angiogenesis and tumor growth via blocking VEGFR2 signaling pathway
Source: Heliyon. 2023 Jun 7;9(6):e17072. doi: 10.1016/j.heliyon.2023.e17072 (PMC10361242; doi:10.1016/j.heliyon.2023.e17072)
Supplement: Multimedia component 1 [file mmc1.docx]

**
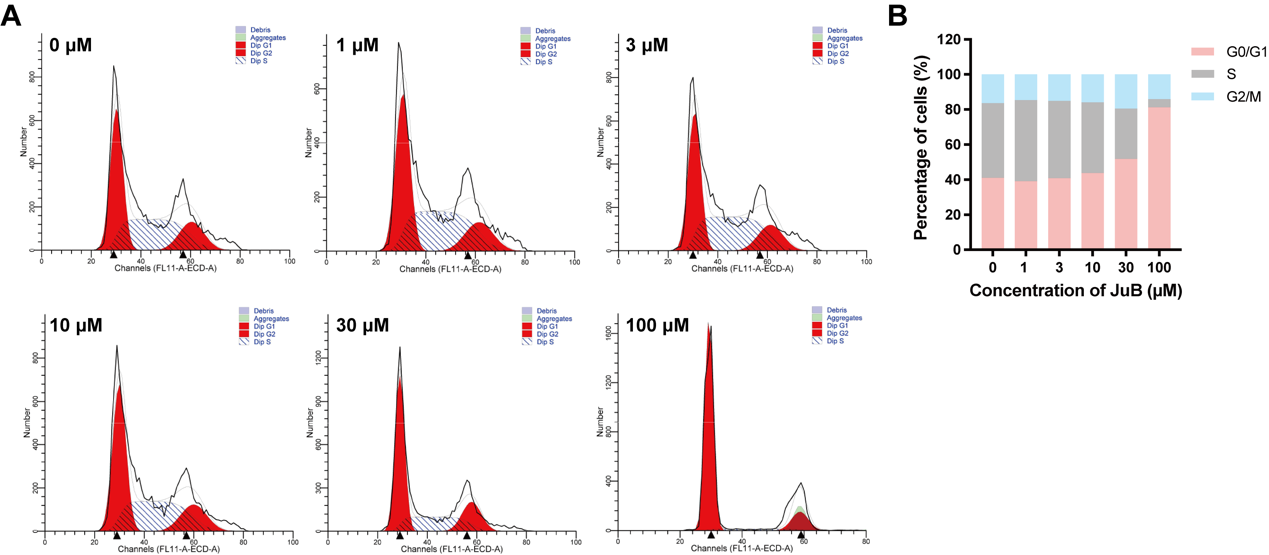
**

**Fig. S1.** Cell cycle analysis of HUVEC cells after JuB treatment. (**A**) Cell cycle profile of HUVEC cells treated with JuB (0, 1, 3, 10, 30, and 100 µM) for 24 h. (**B**) Percentage of cells in each cell cycle phase.

**
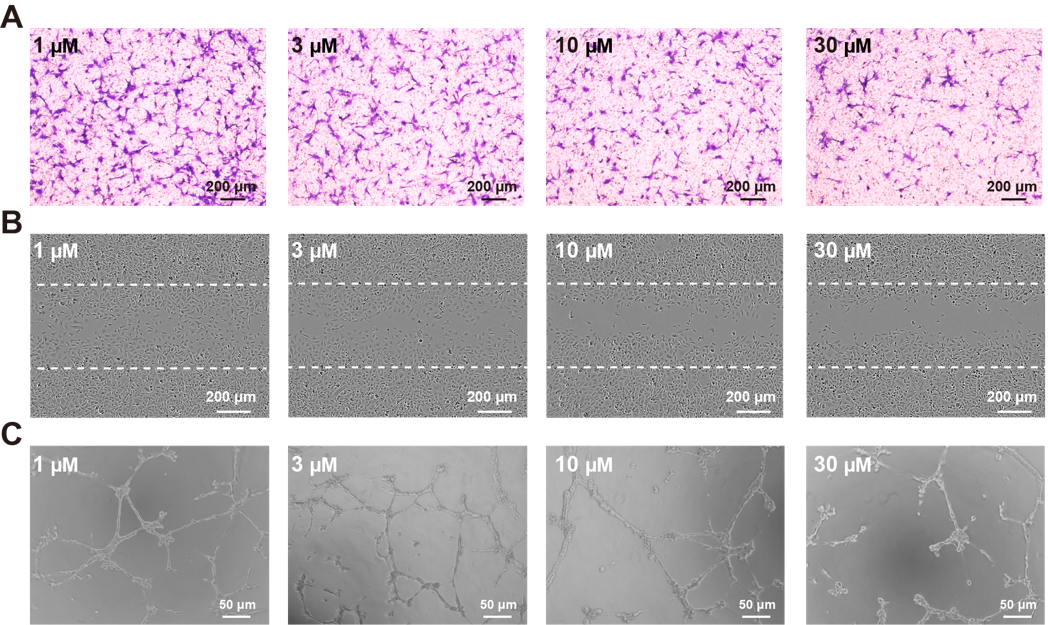
**

**Fig. S2**. JuB significantly inhibited migration and tube formation of HUVECs. (**A**) HUVEC migration was suppressed by JuB in the Transwell assay. (**B**) JuB inhibited the horizontal migration of HUVECs in the wound healing assay. (**C**) JuB suppressed HUVEC tube formation. The photographs of JuB groups (1, 3, 10, 30, 100 μM) were displayed in each panel.

p-VEGFR2- 230 kDa VEGFR2- 210, 230 kDa


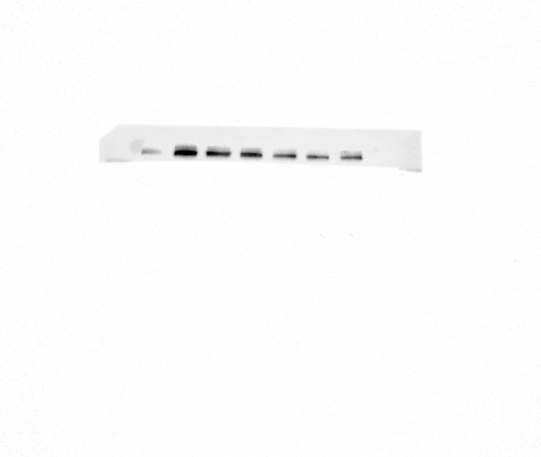

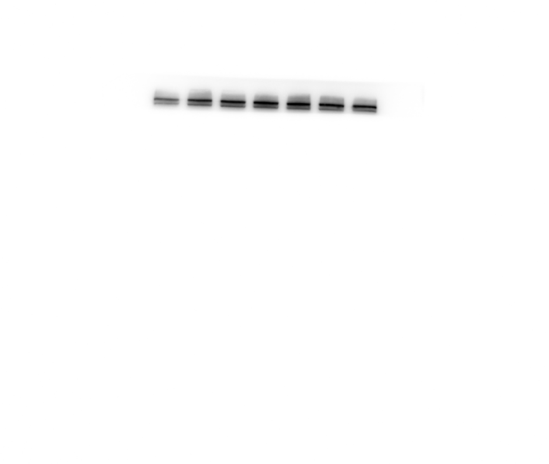


p-PLCγ1- 155 kDa PLCγ1- 155 kDa


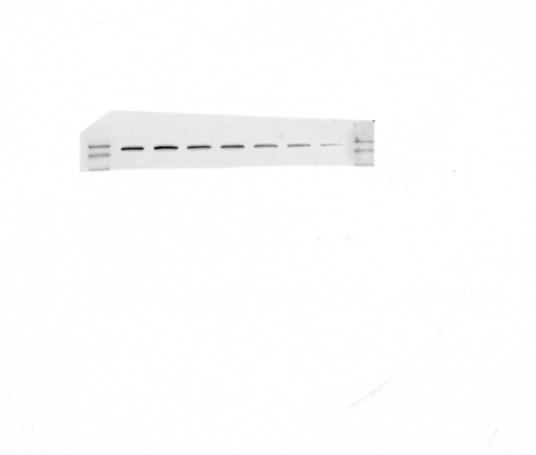

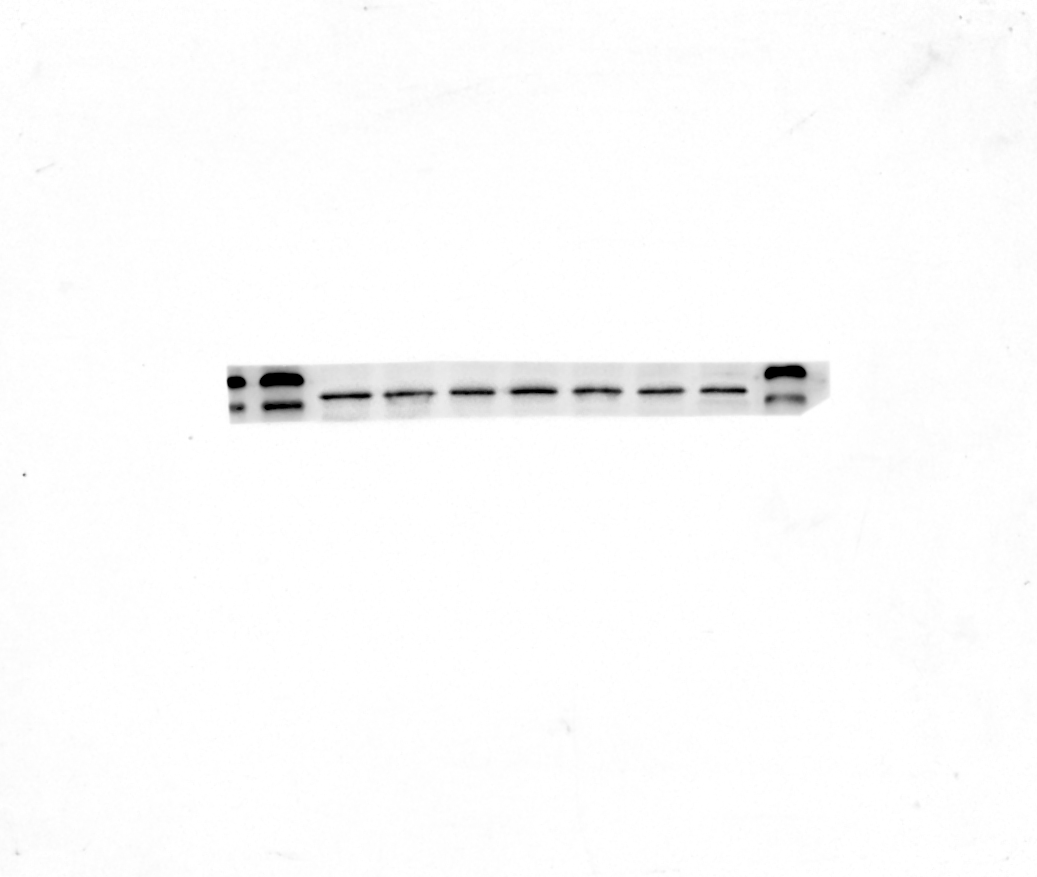


p-Src- 60 kDa Src- 60 kDa


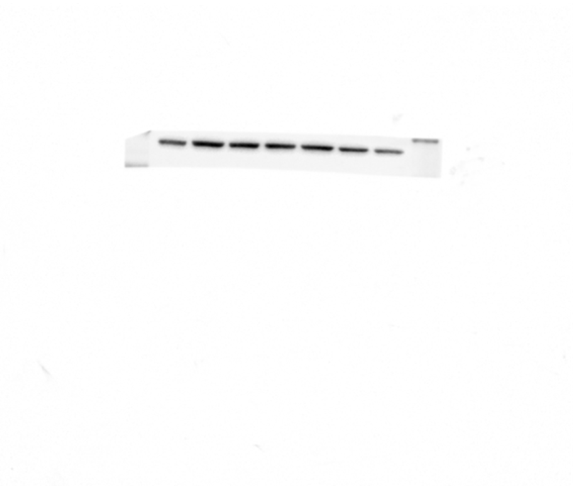

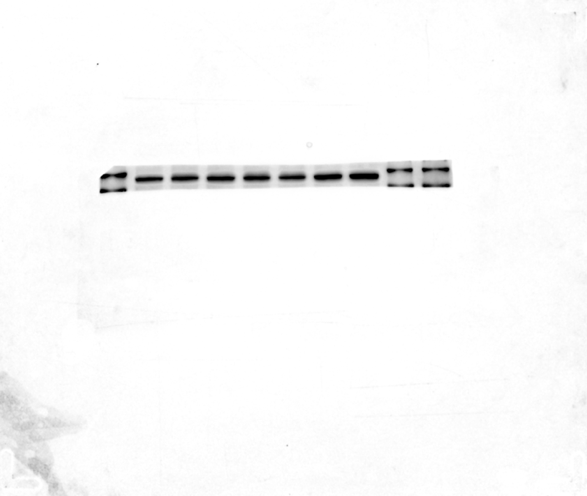


p-FAK- 125 kDa FAK- 125 kDa


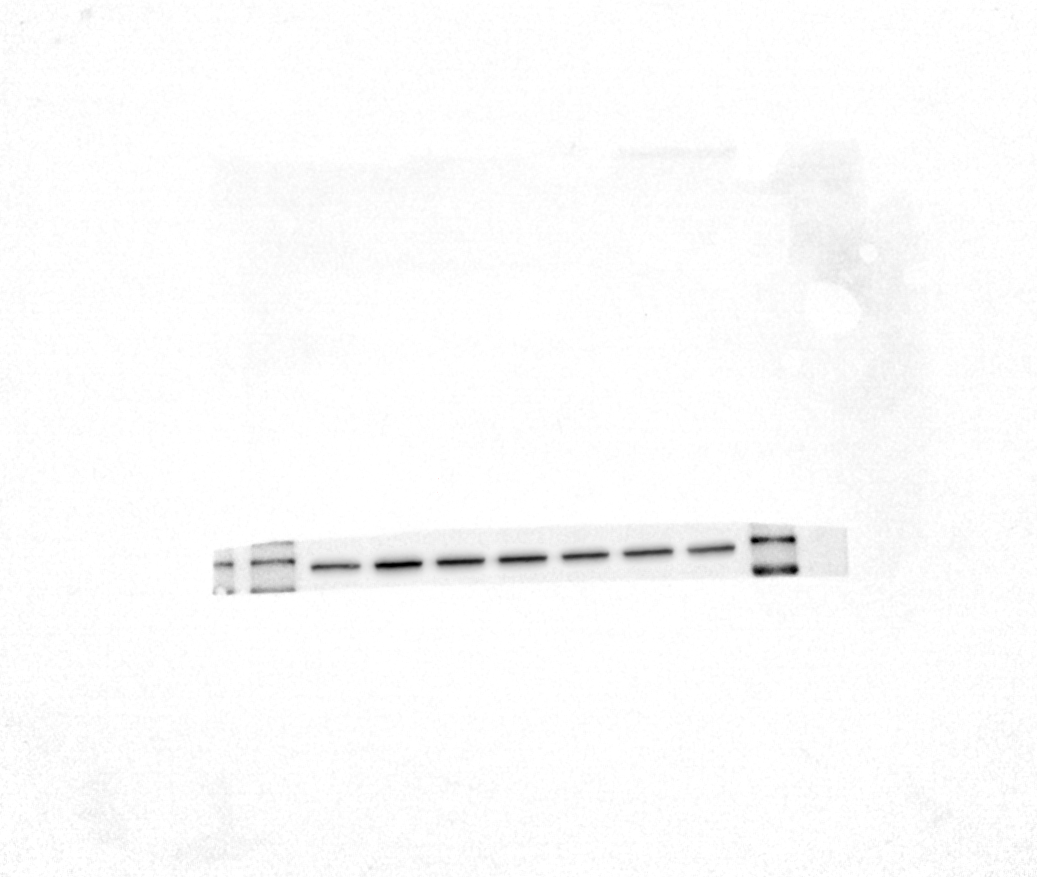

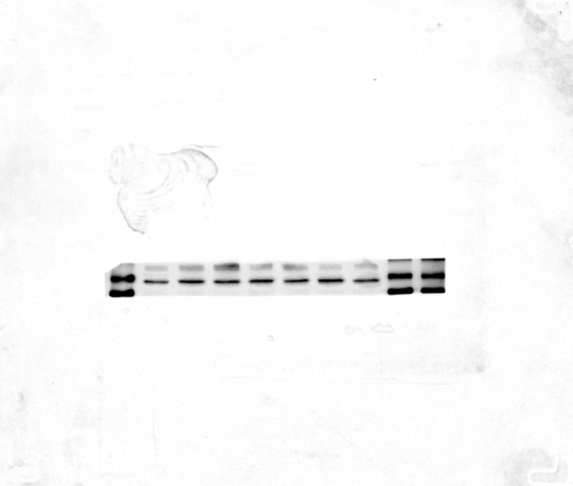


p-Akt- 56 kDa Akt- 56 kDa


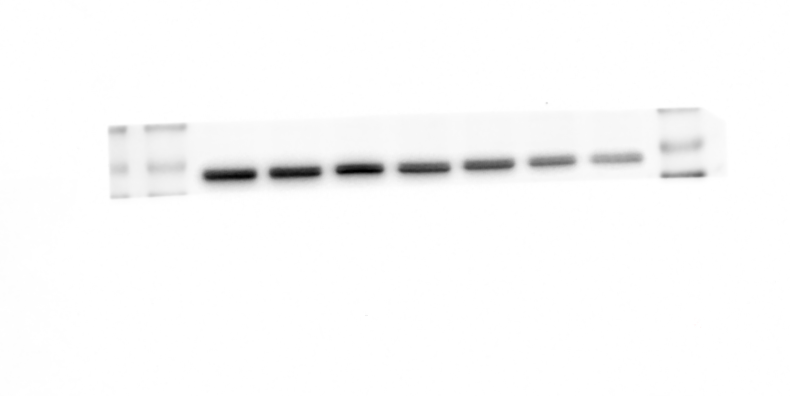

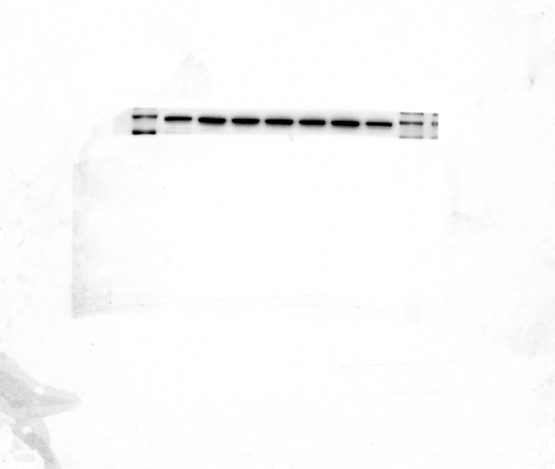


**Fig. S3.** The uncropped images in the western blot assay.


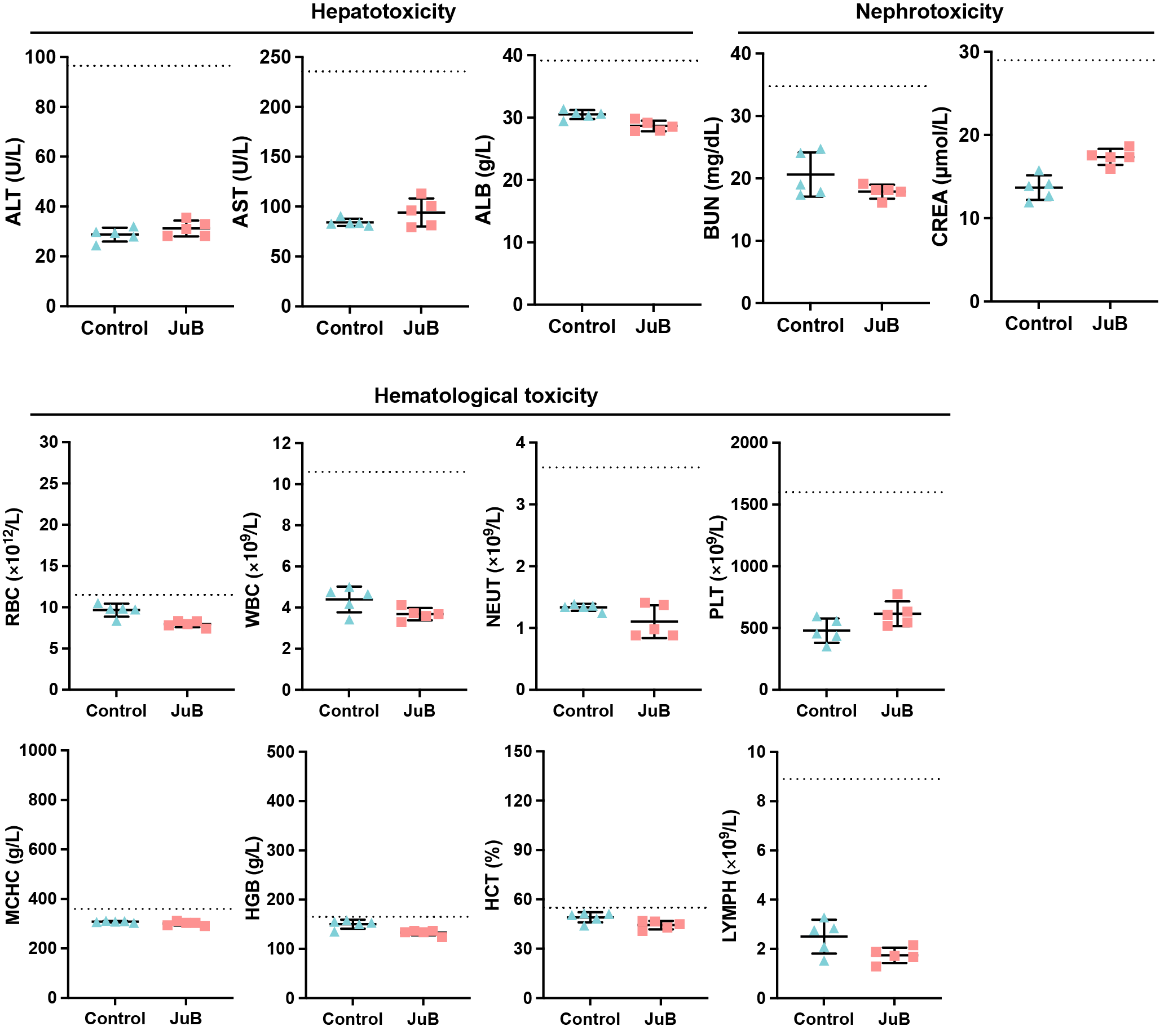


**Fig. S4.** Toxicity profile of JuB in healthy female BALB/c nude mice. The mice were intraperitoneally injected with JuB (20 mg/kg) once every two days for 7 times. 24 h after the last injection, the blood was collected from the mouse orbital for serum biochemistry and complete blood panel analysis. The dotted line indicates the normal ranges of the parameters. ALT, alanine aminotransferase. AST, aspartate aminotransferase. ALB, albumin. BUN, blood urea nitrogen. CREA, creatinine. RBC, red blood cells. WBC, white blood cells. NEUT, neutrophils. PLT, platelets. MCHC, mean corpuscular hemoglobin concentration. HGB, hemoglobin. HCT, hematocrit. LYMPH, lymphocytes. Data are presented as mean ± s.d. (n=5).
